# 3D Printing of Oxygen-Sensing ECM-Based Skin Graft for Personalized Treatment of Chronic Wounds—A Technological Proof of Concept

**DOI:** 10.3390/jfb17010028

**Published:** 2026-01-01

**Authors:** Yehonatan Zur, Rotem Hayam, Nir Almog, Inna Kovrigina, Limor Baruch, Aharon Blank, Marcelle Machluf

**Affiliations:** 1 The Laboratory for Cancer Drug Delivery & Cell Based Technologies, Faculty of Biotechnology & Food Engineering, Technion—Israel Institute of Technology, Haifa 32000, Israel; 2 Schulich Faculty of Chemistry, Technion—Israel Institute of Technology, Haifa 32000, Israel

**Keywords:** skin graft, extracellular matrix (ECM), electron spin resonance (ESR), oxygen sensor, additive manufacturing, 3D bioprinting, tissue engineering

## Abstract

Chronic diabetic wounds are often characterized by persistent hypoxia and poor healing outcomes, highlighting the need for regenerative grafts that not only promote tissue repair but also provide insights into the wound microenvironment. In this study, we introduce a novel strategy for diabetic ulcer treatment through the development of a structurally personalized skin graft. The graft is fabricated via 3D bioprinting of natural porcine skin extracellular matrix (psECM) and integrated with microsensors for oxygen monitoring. We established a porcine skin decellularization protocol that efficiently removed cellular components, while preserving the integrity of the ECM, as verified by DNA quantification and scanning electron microscopy. The resulting psECM bioink demonstrated rheological properties suitable for 3D printing, which depended on psECM concentration and exhibited temperature-responsive gelation behavior. Incorporation of LiNC-BuO oxygen microsensors into the bioink enabled real-time, non-invasive oxygen level monitoring within the printed constructs. Both in vitro and in vivo studies confirmed the cytocompatibility and low immunogenicity of the psECM-based grafts with embedded microsensors. Moreover, the 3D bioprinting technology enabled the manufacturing of grafts tailored to match individual wound geometries. The technological proof of concept presented herein for this multifunctional platform, which integrates the regenerative benefits of ECM scaffolds with advanced biosensing capabilities, represents a promising approach for enhancing future therapeutic outcomes in the management of diabetic ulcers.

## 1. Introduction

Diabetic ulcers, a common and severe complication of diabetes mellitus, pose a substantial global healthcare burden [1]. These persistent, non-healing wounds primarily afflict the lower extremities of diabetic individuals. Left untreated, diabetic ulcers can lead to severe complications such as infections, gangrene, and even the amputation of limbs [2]. Hence, timely and effective treatment is paramount for preventing further complications and enhancing patients’ quality of life. While traditional methods like advanced wound dressings, negative pressure wound therapy, and wound debridement are commonly used, diabetic ulcer healing remains a frustrating and unpredictable issue, often resulting in extended treatment periods and recurrent wound problems [3,4]. The underlying mechanisms that impede wound healing in diabetes are intricate and not yet fully understood. Chronic inflammation, oxidative stress, abnormal growth factor signaling, and compromised blood supply all contribute to the chronicity of diabetic ulcers [5,6,7]. Adequate oxygenation is a fundamental requirement for cellular metabolism, collagen synthesis, and the overall orchestration of wound repair mechanisms. Thus, diabetic ulcers frequently face hypoxia, a deficiency in oxygen, due to compromised blood flow in those regions, hindering the repair of tissues [8]. Currently, no direct method is clinically used to measure oxygen levels within the wound. In the clinic, the gold standard involves using indirect approaches to assess oxygen diffusion from the skin to its surface [9,10]. To tackle these challenges in diabetic ulcer therapy, innovative approaches in regenerative medicine have emerged as potential solutions, through the development of skin grafts, sustained-release targeted drug therapies, and cell therapies. Skin grafts that are based on synthetic materials offer tunable mechanical and structural properties as well as affordable costs but remain biologically inert, frequently showing risks of foreign-body reactions or suboptimal remodeling [11,12,13]. On the other hand, skin grafts based on extracellular matrix (ECM) derivatives, such as acellular dermal matrices, are widely used in the clinic and generally yield superior results compared with traditional wound care [14,15]. The ECM is a complex and dynamic network of proteins crucial for maintaining the integrity and function of all tissues. It consists primarily of fibrous proteins like collagen and elastin, as well as glycoproteins such as glycosaminoglycans (GAGs), proteoglycans, fibronectin, and laminin [16,17]. In tissues, the ECM undergoes continuous remodeling, a process involving the interplay of anabolic and catabolic enzymatic activities by its residing cells. This remodeling is essential for tissue development, wound healing, and regeneration, allowing tissues to adapt to changing needs and repair injuries [18]. Given its tissue-specific composition and diverse functions, the ECM stands out as an attractive biomaterial for regenerative medicine and tissue engineering [17,19,20]. ECM from porcine origin is particularly suitable for grafts because its structural and bioactive components closely match those of human ECM, reflecting strong cross-species conservation. These molecular similarities support comparable immune responses and remodeling outcomes to those observed with human-derived scaffolds. Pigs also provide a scalable and standardized tissue source due to their size, physiology, and rapid breeding, making porcine ECM both practical and reliably available for clinical use [16,21].

Though offering a significant improvement over traditional wound care, current ECM-based skin grafts often show inconsistent outcomes, with limited mechanical strength and incomplete integration, and they lack the capacity to precisely match wound geometry or provide dynamic biological feedback [14,15]. A structural fit to the wound’s geometry holds the potential to significantly enhance the skin graft’s therapeutic efficacy, particularly in complex anatomical regions or irregularly shaped wounds. To address this challenge, we propose utilizing 3D bioprinting technology in the fabrication of porcine ECM grafts that structurally fit each wound. Three-dimensional bioprinting, an advanced additive manufacturing technology, enables the precise production of computer-aided designed three-dimensional structures using diverse bioinks [22,23]. Bioinks are biomaterials that possess rheological properties such as viscoelastic behavior, yield stress, and gelation point, which enable smooth extrusion and shape fidelity during the 3D printing process [23]. Hydrogels, known for their biocompatibility and ability to mimic the extracellular matrix of tissues, are commonly used as bioinks, as they maintain cell viability and structural functionality during and after the 3D bioprinting process [22,24,25,26]. In this process, the 3D printer dispenses the bioink layer-by-layer to construct a 3D model. Precise control of the printing nozzle and bioink flow rate is crucial to ensure the accurate deposition of the bioink.

Our goal in the current research was to develop personalized ECM-based skin grafts that not only offer a personalized structural fit but also sense and report the oxygen levels in the healing ulcer. To this end, we developed a bioink composed of porcine skin ECM (psECM) integrated with lithium octa-n-butoxynaphthalocyanine, also known as LiNC-BuO [27]. This unique compound serves as a paramagnetic naphthalocyanine complex and is used as an oxygen microsensor, read by electron spin resonance (ESR) [28]. ESR is a powerful spectroscopic method that enables the detection and quantification of free radicals and paramagnetic species, including molecular oxygen. In our application, we make use of the fact that the relaxation time, denoted *T*_2_, of the paramagnetic material is inversely proportional to the oxygen partial pressure [29]. We hypothesized that by introducing oxygen microsensors into the printed psECM grafts, these grafts would allow real-time ESR monitoring of oxygen dynamics within the ulcer bed. This information can be pivotal for clinicians and researchers in the future, offering insights into the extent of tissue hypoxia, the effectiveness of the therapeutic intervention, the overall progression of wound healing, and the necessity for complementary oxygen enrichment therapies.

## 2. Materials and Methods

Skin ECM hydrogel: Porcine skin tissues of healthy slaughter-weight pigs were purchased from LAHAV C.R.O. (Lahav, Israel). Skin decellularization protocol was adapted from our previously published work [30,31,32] with some modifications. Briefly, excess fat was removed, and the skin was cut into 0.5 × 0.5 cm pieces, which were then decellularized using alternating hyper/hypotonic NaCl solutions, enzymatic digestion using trypsin-EDTA (Merck, Darmstadt, Germany), and detergent washes using 1% Triton^®^-X-100 (Merck) and 0.1% ammonium hydroxide in PBS. To produce psECM bioink, acellular psECM was frozen in liquid nitrogen and lyophilized. The lyophilized psECM was then solubilized (25–30 mg mL^−1^) in HCl (0.01 M) using pepsin enzymatic digestion (1 mg mL^−1^). The solution was then adjusted to pH = 6 with NaOH and kept cold (4 °C).

DNA quantification: DNA was extracted from native and decellularized skin samples using TRI Reagent (Merck), following the manufacturer’s instructions. Quantification of the extracted DNA was performed using the PicoGreen™ dsDNA assay (ThermoFisher Scientific, Waltham, MA, USA) in accordance with the manufacturer’s protocol.

SEM analyses: Lyophilized psECM samples were analyzed by scanning electron microscopy (SEM) using a Zeiss Ultra-Plus FEG-SEM. EHT = 2 kV; aperture size = 30 µm.

Rheological analyses: The viscoelastic properties of psECM bioinks were characterized using a DISCOVERY HR-2 Hybrid Rheometer (TA Instruments, New Castle, DE, USA). For gelation kinetics, 0.5 mL of solubilized psECM was loaded onto a Peltier plate pre-equilibrated at 4 °C. The temperature was then rapidly increased to 37 °C to initiate gelation, and oscillatory time-sweep measurements were conducted over a 30 min period at a fixed angular frequency of 1 rad/s and a strain amplitude of 1%. Gelation progression was monitored by tracking the evolution of the storage modulus (G′) over time. For frequency-sweep analysis, fully gelled samples were subjected to oscillatory deformations across a range of angular frequencies (0.1–600 rad/s) to assess the material’s viscoelastic behavior under varying timescales. Measurements were performed at 37 °C using a parallel-plate geometry (40 mm diameter, 1.2 mm gap) under 1% strain, ensuring that deformation remained within the linear viscoelastic range.

Thermal gravimetric analysis: Samples of psECM bioink crosslinked with 0.2–0.5% EGCG were lyophilized and subjected to thermogravimetric analysis (TGA) using a TGA-Q5000 system (TA Instruments). Samples were heated from room temperature to 600 °C at a rate of 20 °C/min under a constant nitrogen atmosphere. Weight loss data were recorded continuously and analyzed using Trios TA Universal Analysis 200 Software version 4.5A build 4.5.0.5 (TA Instruments).

Support bath preparation: To enable stable 3D bioprinting of soft psECM-based hydrogels, we employed a support bath composed of low-acyl gellan gum (Kelcogel F, Modernist Pantry, York, ME, USA). A 0.5% (*w*/*v*) gellan gum solution was prepared in PBS containing 0.1% (*w*/*v*) calcium chloride dihydrate (Merck) and heated in a boiling water bath for at least 20 min to ensure full dissolution. Following cooling to room temperature, the gelled solution was mechanically processed to generate microparticles. This was achieved either by passing the bulk hydrogel through a stainless-steel mesh with 100 μm pores or by homogenization using a Precellys 24 tissue homogenizer (Bertin Instruments, Montigny-le-Bretonneux, France) operated at 4000 rpm for 10 s. Both methods produced consistently fragmented support material suitable for bioprinting applications.

Gellan gum particles’ size distribution: The size distribution of the gellan gum microparticles was characterized using laser diffraction with a Mastersizer 3000 particle size analyzer (Malvern Instruments Ltd., Worcestershire, UK). Measurements were based on Mie and Fraunhofer light-scattering models as computed by the instrument’s software. Particle size was reported using volume-weighted percentiles, specifically Dv10 (10% of particles below this size), Dv50 (median particle size), and Dv90 (90% of particles below this size). Measurements were conducted in five independent replicates (n = 5).

Ulcer shape imaging and modeling: Cutaneous ulcers were imaged at the Department of Internal Medicine, Rambam Health Care Campus (Haifa, Israel), under Helsinki Committee approval. A commercial handheld structured-light scanner (EinScan H2, SHINING 3D, Hangzhou, China) was used. Patients were scanned in bed while the operator moved the scanner around the affected limb to capture complete 3D surface geometry (nominal spatial sampling ~0.5 mm). The acquired data was reconstructed with the manufacturer’s software and exported for downstream analysis.

Three-dimensional printing process: psECM was printed using a Bio X 3D printer (Celllink, Göteborg, Sweden), with a stereolithography file format built into the printer database. 3 mL samples of bioink were placed in a 3 mL luer lock syringe (BD Medical Surgical, Franklin Lakes, NJ, USA) and transferred to the printer’s tubes. The 3D printing was conducted at a temperature of 20 °C using a 25G nozzle, an extrusion pressure of 140 kPA, and an extruder moving speed of 6.5 mm/s. The 3D-printed objects were printed inside 6-, 12-, or 24-well plates filled with gellan gum (GG) support bath. Crosslinking was performed by adding epigallocatechin gallate (EGCG) to the GG bath at a final concentration of 0.03% and incubating for 24 h.

Stability tests: Sample stability was measured by weight loss over time. Bioprinted psECM constructs (5 mm × 5 mm × 5 mm) crosslinked with EGCG (0.03% or 0.1%) were incubated in 2 mL double-distilled water in 12-well plate for 30 days. Samples were weighed on day one and every seven days thereafter. The results of each measurement were derived from at least three independent samples.

Mechanical properties: Mechanical properties were assessed using displacement-controlled compression at 37 °C with a microscale testing system (MicroTester G2, CellScale, Waterloo, ON, Canada). Bioprinted psECM constructs (5 mm × 5 mm × 5 mm) crosslinked with EGCG (0.015% or 0.03%) were subjected to 10% displacement using a 0.4 mm diameter microbeam attached to a 6 × 6 mm compression plate. Each specimen underwent a 60 s loading phase followed by a 30 s recovery period, and the cycle was repeated once. Force–displacement data were recorded throughout, and Young’s modulus was calculated from the loading curves (n ≥ 4).

Integration of oxygen microsensors in the psECM skin graft: To incorporate oxygen sensors into the printed graft, 5 mm × 5 mm × 5 mm cubes were fabricated using a dual-syringe 3D bioprinting setup. One syringe was filled with standard bioink to print the bulk of the cube, while the second syringe contained bioink mixed with LiNc-BuO oxygen microsensors (a generous gift from Prof. Periannan Kuppusamy, Dartmouth College) at a concentration of 10 mM. During printing, the sensors-containing bioink was precisely deposited—10 μL in total—into the center of the cube at two specific depth options: 0.5 mm and 1 mm from the outer edge. This approach allowed spatially defined integration of sensors within the construct.

ESR oxygen measuring within the printed graft: ESR oxygen measurements within the printed graft were performed using a pulsed ESR spectrometer (spinUP-S, SpinFlex Ltd., Ashkelon, Israel) operating in the 2–4 GHz band. This system transmits short microwave pulses that excite the electron spins in the paramagnetic material and then measures the signal as it decays after the excitation pulses. This exponential decay is characterized by a time constant, $T_{2}$, which is inversely proportional to the oxygen partial pressure ($pO_{2}$). Following calibration under standard conditions of 0, 1, 4, 16, and 22% oxygen, one can extract the local $pO_{2}$ at the site of the paramagnetic sensors from the measured $T_{2}$ [33]. The instrument was coupled to a custom compact ESR scanner comprising a small permanent-magnet array and a miniature microwave resonator. ESR signals from the paramagnetic particulates embedded in the skin graft were acquired and analyzed to infer oxygen levels. Full details of the experimental setup are provided elsewhere [34].

Cell culture: The immortalized human keratinocyte cell line HaCaT was cultured in DMEM (Merck) supplemented with 10% FBS (Biowest, Nuaillé, France), 2 mM L-glutamine (Gibco, ThermoFisher Scientific, Waltham, MA, USA), and 1% penicillin–streptomycin. NIH/3T3 murine fibroblasts (CRL-1658™, ATCC) were cultured in DMEM containing 10% FBS and 1% penicillin–streptomycin. All cell lines were maintained at 37 °C in a humidified incubator with 5% CO_2_. The murine macrophage cell line RAW 264.7 (TIB-71™, ATCC, Manassas, Virginia) was maintained in Dulbecco’s Modified Eagle Medium (DMEM; Merck) supplemented with 10% fetal bovine serum (FBS; Biowest) and 1% penicillin–streptomycin (Biowest). Medium was replaced every two days.

Cytocompatibility: HaCaT and 3T3 cells were co-seeded at a 4:1 ratio, with a total of 100,000 cells per well, in a 48-well plate. Four experimental conditions were tested based on the well surface: Control—standard wells with no modification; Bioink—wells coated with a layer of bioink on the bottom surface; Bioink + Microsensors (5 mM and 10 mM). Cell viability was assessed one day post-seeding using the AlamarBlue™ assay (AbD Serotec, Kidlington, UK), according to the manufacturer’s instructions. Results were normalized to the viability values of the control group on day 1. Subsequent viability measurements were performed on days 3, 7, 10, and 14, with the medium replaced after each time point. Additionally, Live/Dead cell analysis using fluorescein diacetate (FDA) and propidium iodide (PI) (Merck) were conducted on days 7 and 14 to qualitatively evaluate cell viability. Fluorescent imaging was performed using a Nikon TE2000-E microscope (Nikon, Tokyo, Japan).

In vitro immunogenic potential: The RAW macrophage cell line was cultured for 24 h in a low serum-containing medium (2% FBS). Then, cells were exposed to 20 mg of lyophilized psECM, lyophilized bioink, LiNc-BuO particles, or PLGA (Merck). As a positive control, lipopolysaccharide 1 μg/mL (LPS, Merck) was used, and as a negative control (basal), untreated cells were used. After 16 h of stimulation, the secretion of nitric oxide (NO) was measured using the Griess Reagent System (Promega, Madison, WI, USA), according to the manufacturer’s protocol. The expression levels of the pro-inflammatory cytokine IL-1β, were quantified using real-time RT-PCR with the following specific primers:

5′-AGGATGAGGACATGAGCACC-3′ and 5′-ATGGGAACGTCACACACCAG-3′.

RNA was extracted using TRI reagent (Merck) according to the manufacturer’s protocol and reverse-transcribed in a PTC-200 PCR cycler (MJ Research, Reno, Nevada, USA) using a qScript cDNA synthesis kit (Quantabio, Beverly, MA, USA).In vivo immunogenic potential: Animal experiments were approved by the Animal Ethics Committee at the Technion, Israel. Six-week-old male C57BL mice were anesthetized using 2.5% isoflurane, and subcutaneously injected with psECM bioink, psECM bioink integrated with oxygen microsensors (10 mM), or alginate hydrogel as a negative control (n = 6 mice per group per time point, Harlan Labs, Jerusalem, Israel). No postoperative analgesia was used. Animals were euthanized using CO2 asphyxiation at 7 or 21 days post-implantation, and blood samples were taken for cytokine quantification. Systemic cytokine levels were assessed using the Mouse Cytokine/Chemokine Magnetic Bead Panel (Millipore, Burlington, MA, USA), targeting TNF-α, IL-1β, IL-6, and IFN-γ. Following explantation, the injected hydrogel constructs were harvested and processed for histological evaluation. Samples were fixed, embedded in paraffin blocks, sectioned at 5 μm thickness using a Leica RM2255 or RM2235 microtome (Wetzlar, Germany), and stained with hematoxylin and eosin (H&E) for general histopathological assessment. To evaluate immune cell infiltration, immunohistochemical (IHC) staining for macrophages was performed using an anti-F4/80 antibody (1:100 dilution; Serotec, Oxfordshire, UK, MCA479R).

Statistical analysis: All data are expressed as mean ± standard deviation (SD) based on at least three independent replicates for each experimental group and time point. Statistical differences between groups were evaluated using unpaired two-tailed Student’s *t*-tests unless otherwise indicated. A *p*-value < 0.05 was considered statistically significant.

## 3. Results

### 3.1. Porcine Skin Decellularization

**Figure 1:**
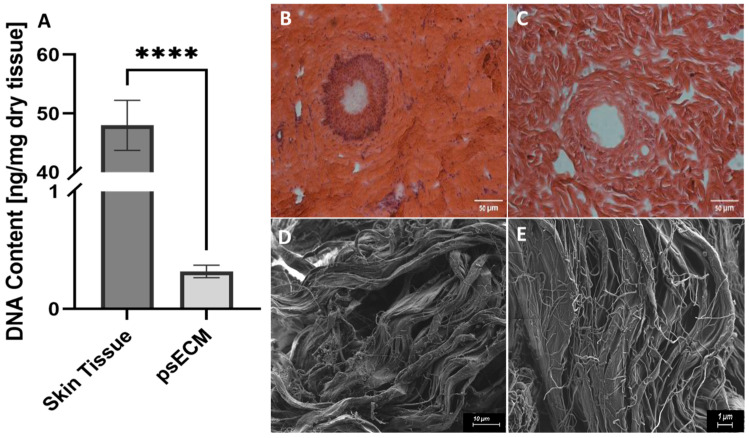
Characterization of psECM. (**A**) DNA content in porcine skin extracellular matrix (psECM) compared with native porcine skin tissue. (**B**,**C**) H&E histological analyses of skin tissue (**B**) compared with psECM (**C**). (**D**,**E**) Scanning electron microscopy (SEM) analysis revealing the structural properties of psECM. (**B**,**C**) Scale bars: 50 µm; (**D**) scale bar: 10 µm; (**E**) scale bar: 1 µm. **** *p* < 0.0001.

The first step in developing a psECM-based skin graft was to develop a decellularization protocol for skin tissue while avoiding harsh detergents such as sodium dodecyl sulfate (SDS). In this process, we relied on our previous work on ECM isolation from different porcine tissues [21,30,31] and modified it to optimally decellularize skin tissue. To confirm the effectiveness of the developed decellularization protocol, we quantified the DNA that remained in the psECM and compared it with native skin tissue, revealing a minimal presence of less than 0.5 ng per mg of dry tissue [26] (Figure 1A). Histological analyses confirmed these findings, demonstrating the high DNA content in native porcine skin tissue (Figure 1B), in contrast to psECM, in which no visible DNA was detected (Figure 1C). To further characterize the obtained psECM, SEM analysis was performed, depicting a porous network of fibers of varying sizes (Figure 1D,E).

### 3.2. Development of psECM Bioink

**Figure 2:**
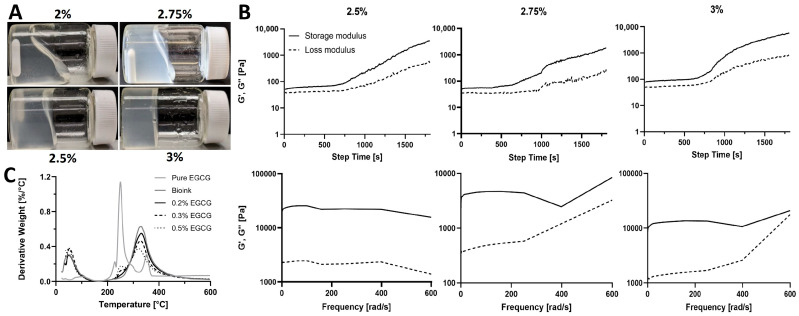
psECM bioinks. (**A**) Bioinks of different psECM concentrations. (**B**) Upper panel: Time-sweep rheological characterization of different psECM concentration bioinks, showing changes in storage (G′) and loss (G″) moduli over time. Lower panel: Frequency-sweep rheological characterization of different psECM concentration bioinks, showing changes in storage (G′) and loss (G″) moduli in ascending frequencies. N = 4. (**C**) Thermogravimetric analysis (TGA) of psECM bioinks crosslinked using varying concentrations of epigallocatechin gallate (EGCG).

To produce psECM bioinks capable of thermally induced gelation, lyophilized decellularized porcine skin ECM (psECM) was enzymatically digested using pepsin and solubilized at varying concentrations (2%, 2.5%, 2.75%, and 3% *w*/*v*, Figure 2A). The rheological behavior of the resulting bioinks was evaluated by time-sweep and frequency-sweep analyses to characterize gelation kinetics and viscoelastic properties. In the time-sweep analysis, rheological measurements were performed at a constant temperature of 37 °C following a temperature rise from 4 °C, simulating physiological conditions for gelation. For all three concentrations, both the storage modulus (G′) and loss modulus (G″) increased over time, with G′ consistently exceeding G″ throughout the measurement. This early dominance of G′ indicates that the bioinks rapidly acquired solid-like viscoelastic properties. However, none of the concentrations exhibited a clear plateau in G′ within the initial 30 min period, suggesting that gelation was still ongoing (Figure 2B). A similar trend was observed for the 2.75% formulation extended to 60 min, in which G′ continued to rise, indicating progressive network stabilization (Figure S1). Frequency-sweep analysis was performed to assess the response of the gelled hydrogels to oscillatory deformations across a range of angular frequencies (0.1–400 rad/s). In all samples, G′ remained greater than G″ throughout the frequency range, and both moduli showed mild frequency dependence, characteristic of a physically crosslinked, weak gel-like material [35]. At higher frequencies, a slight decline in G′ was observed, indicating limitations in the material’s ability to maintain structural integrity under rapid deformation (Figure 2B). This behavior likely reflects the onset of network relaxation rather than a mechanical failure or yield point and is typical for collagen-based hydrogels with transient physical interactions [36]. Based on these analyses, the 2.75% psECM concentration was selected for further development. This concentration offered a favorable balance between mechanical robustness and processing practicality. While the 3% formulation exhibited higher stiffness, its preparation required extended digestion and neutralization times, which posed significant challenges in reproducibility and workflow. In contrast, the 2.75% psECM formulation achieved faster solubilization and thermal gelation readiness, making it a more practical choice for scalable bio fabrication. In addition to the temperature-induced gelation, we examined the addition of a crosslinker to the bioink (2.75% psECM) to further enhance the mechanical strength and stability of the formed hydrogel. Our crosslinker of choice was EGCG, a phenolic compound that is the most abundant and active antioxidant component in tea polyphenols. With its hydroxyl groups, EGCG is able to form covalent and non-covalent bonds with biological macromolecules such as proteins. It is biocompatible and considered safe, making it suitable for biomedical applications [37,38,39]. Moreover, EGCG exhibits fast crosslinking kinetics, offering quick gelation post-bioink deposition and consequently high printing resolution and easy handling of the printed models. Through thermal gravimetric analysis (TGA), we assessed the effect of EGCG addition (0.2–0.5%) on the thermal decomposition of the psECM bioink, expressed as the first derivative of weight loss as a function of temperature (Figure 2C). The non-crosslinked bioink was characterized as two peaks at 50 °C and 330 °C, typical of collagen decomposition [40,41]. An additional peak was observed in the crosslinked psECM bioinks at 250 °C, corresponding to the prominent peak in the EGCG decomposition profile. As expected, this peak increased with increasing EGCG concentration. At 330 °C, on the other hand, the trend was reversed, with the bioink demonstrating the highest decomposition peak and the 0.5% EGCG bioink sample showing the lowest.

### 3.3. 3D Bioprinting of Structurally Fitted psECM Skin Grafts

**Figure 3:**
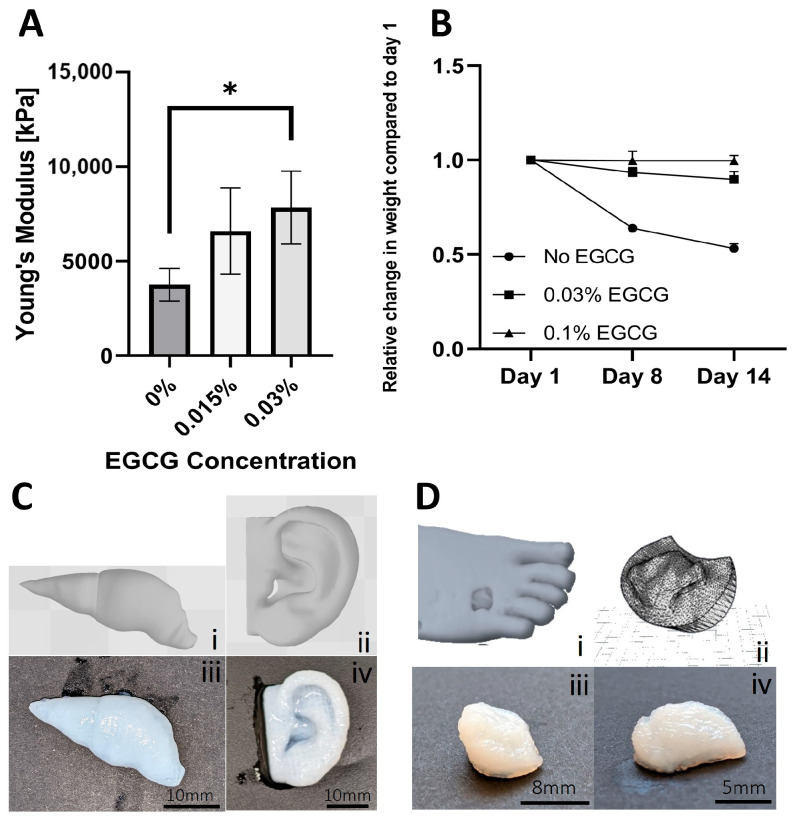
Three-dimensional bioprinting of psECM. (**A**) Mechanical properties of 5 mm × 5 mm × 5 mm cube model printed from psECM and crosslinked using different EGCG concentrations. N = 5. * *p* < 0.05. (**B**) Construct stability of 5 mm × 5 mm × 5 mm printed psECM models during 14 days of incubation in double-distilled water. (**C**) Models of a liver (**i**) and an ear (**ii**) and their corresponding printed specimens (**iii**,**iv**). (**D**) Three-dimensional bioprinting of a patient’s diabetic ulcer. (**i**) Scanned diabetic ulcer. (**ii**) CAD model of the scanned ulcer, and (**iii**,**iv**) printed models from psECM bioink.

After the bioink formulation and rheological properties were developed and evaluated, the next step involved the optimization of the printing method. An inherent limitation of extrusion 3D printing onto a surface is the deformation and collapse of the extruded soft bioinks. One way to address this problem is by using a support bath [42,43], whose primary function is to stably hold multiple layers of printed material during the printing process while enabling easy removal once this process is completed. In our study, we utilized a gellan gum (GG) support bath [44], which was optimized in terms of particle size distribution to enhance printing resolution and accuracy (Figure S2). Using the optimized support bath, bioprinting of psECM was facilitated. Primarily, a 5 mm × 5 mm × 5 mm cubic model was printed and crosslinked using 0.03% or 0.015% EGCG in GG. To assess the mechanical properties of the printed psECM models, we performed compression tests, in which controlled compressive forces were applied to the printed structures, and their mechanical durability and ability to resist deformation were quantified. Our results revealed no statistically significant difference between the Young’s modulus of printed constructs crosslinked using 0.015% or 0.03% EGCG; however, constructs crosslinked with 0.03% EGCG displayed a significantly higher Young’s modulus compared with models with no EGCG (Figure 3A). To evaluate construct stability, a stability test was conducted, revealing that over a 14-day period, the weight of the 0.1% EGCG-crosslinked samples remained constant. Moreover, no visual deformations of the model’s structure was observed in these models. In the 0.03% EGCG-crosslinked samples, a slight decrease in weight was observed (10%), attesting to their partial dissolution, while the non-crosslinked samples exhibited a 50% decrease in weight after 14 days, with apparent dissolution (Figure 3B).

To demonstrate bioprinting of psECM bioink into more complex shapes, ear and liver models were printed (Figure 3C). Next, to address our capability to produce personalized skin grafts, real patients’ wounds were scanned and converted into CAD models. The grafts, structurally fitted to the scanned wound shapes were then bioprinted from psECM using optimized parameters (Figure 3D).

### 3.4. Integration of Oxygen Microsensors in the psECM Skin Graft

**Figure 4:**
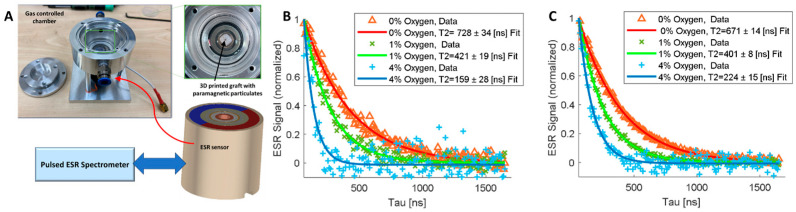
Measuring oxygen levels using a dedicated electron spin resonance (ESR)system [34]. (**A**) Chamber and ESR probe for pO_2_ measurements in a controlled atmosphere. (**B**,**C**) ESR readings of microsensors located at depths of 1 mm (**B**) and 0.5 mm (**C**) from the surface of printed 5 mm × 5 mm × 5 mm cube models. Readings were performed under 0 (red), 1% (green), and 4% (blue) oxygen.

To generate a personalized skin graft that can also detect oxygen levels within the healing wound, we integrated oxygen microsensors (LiNc-BuO) into the graft at a concentration of 5 mM and strategically placed them at specified locations within the printed model. A 5 mm × 5 mm × 5 mm cube model was bioprinted containing oxygen microsensors at depths of 0.5 mm and 1 mm from the surface. Oxygen levels were then read using our recently developed ESR system [34] (Figure 4A) under oxygen levels of 0%, 1%, and 4%. Total measurement time for each decay curve was two minutes. Figure 4B,C exhibit similar trends, with higher oxygen concentrations demonstrating a shorter spin–spin relaxation time (*T*_2_), confirming the ability to measure oxygen levels. Moreover, our analysis revealed distinctions in the quality of readings based on the depth of the microsensors. Microsensors positioned at a further distance from the ESR probe exhibited readings with greater deviations. This discrepancy is evident when comparing the 4% oxygen readings at 1 mm (Figure 4B) with those at 0.5 mm (Figure 4C). The observed differences underscore the sensitivity of the ESR system to variations in sensor depth, providing valuable insights into the spatial dynamics of oxygen concentration within the printed model.

### 3.5. Cytocompatibility of the psECM Oxygen-Sensing Skin Graft

**Figure 5:**
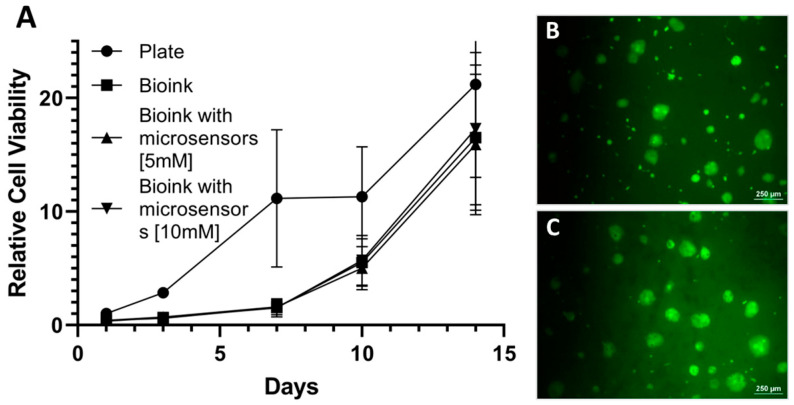
Graft cytocompatibility. (**A**) Relative viability of keratinocytes and fibroblasts cultured on psECM graft with microsensors. N = 5. (**B**,**C**) fluorescein diacetate (FDA)/propidium iodide (PI) live/dead staining of the cultured cells at day 1 (**B**) and day 14 (**C**).

After establishing the technological capability to 3D bioprint psECM skin grafts incorporated with oxygen microsensors and to measure their oxygen levels, the cytocompatibility of the grafts had to be verified. Though our previous studies demonstrated excellent cytocompatibility of porcine ECM from different tissues and LiNC-BuO microsensors proved cytocompatibility in previous studies [45,46], evaluating the specific combination of psECM bioink and microsensors can better predict the graft potential. To this end, we investigated the proliferation of a co-culture of keratinocytes and fibroblast cell lines on the integrated grafts. Figure 5 demonstrates that the viability and proliferation of these cells remained unaffected by the presence of the microsensors. Hence, after two weeks of culture, the number of viable cells was more than 20 times higher than the number of initially seeded cells. As depicted in Figure 5B,C, the cultured cells were viable and their morphology was normal.

### 3.6. psECM Skin Graft Biocompatibility

**Figure 6:**
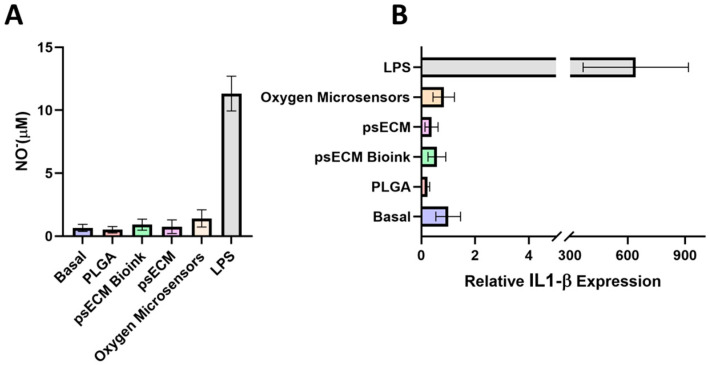
Immunogenic potential of the psECM graft integrated with oxygen microsensors in vitro. (**A**) Nitric oxide (NO)secretion levels after 16 hrs of macrophage excitation. (**B**) Expression of IL-1β mRNA quantified by real-time RT-PCR and normalized to GAPDH.

To assess the immunogenic potential of the developed psECM skin graft integrating oxygen microsensors, we first tested it in vitro through a macrophage excitation assay. RAW macrophages were exposed to psECM, psECM bioink, or oxygen microsensors for 16 h, and their excitation was evaluated through the secretion of nitric oxide (NO) and expression of the pro-inflammatory cytokine IL-1β. In parallel, cells were exposed to LPS as a positive control or to PLGA, an extensively used FDA-approved biodegradable and biocompatible polymer, as a negative control [47]. Figure 6A shows that while high levels of NO were secreted following LPS treatment, the other treatments did not lead to an elevation in the secreted NO levels.

Similar results were obtained for the expression of IL-1β, revealing macrophage excitation following LPS treatment but not following the other treatments, with no significant differences observed between PLGA, psECM, psECM bioink, and oxygen microsensor treatments compared with untreated cells (Figure 6B).

**Figure 7:**
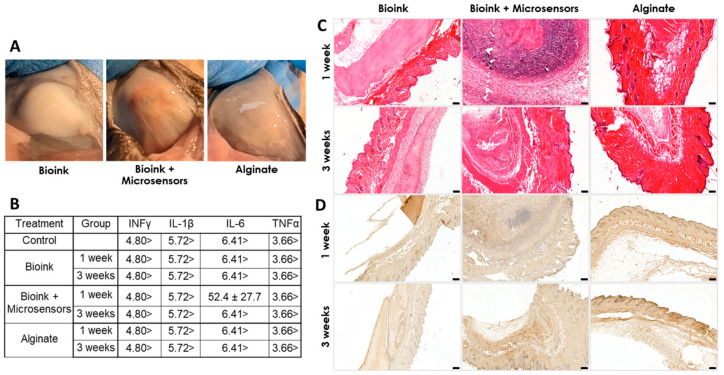
In vivo biocompatibility of the psECM graft integrated with oxygen microsensors. (**A**) Implanted grafts at implantation site after three weeks. (**B**) Blood cytokine levels (INF-γ, TNF-α, IL-1β, and IL-6) one and three weeks post-implantation. Units in pg/mL. N = 6. (**C**) H&E staining of the implants. (**D**) F4/80 macrophage immunostaining of the implants.

We also addressed the biocompatibility of the psECM skin graft containing oxygen microsensors. This was achieved in vivo through subcutaneous implantation in immunocompetent mice. Three different treatments—bioink alone, bioink integrated with oxygen microsensors (5 mM), and alginate, a known biocompatible biopolymer, were subcutaneously injected into mice to evaluate the immune response. Over a three-week monitoring period, the weights of the mice across all treatment groups exhibited similar trends, indicating no discernible impact on animal weight (Figure S3). Upon sacrifice at 7 and 21 days post-implantation, visual inspection of the implantation sites revealed distinct outcomes. While mice injected with bioink alone had no visible vascularization or tissue development around it, mice injected with other treatments had different levels of blood vessel formation (Figure 7A). Analysis of blood cytokine levels revealed undetectable levels of INF-γ, IL-1β, TNF-α, and IL-6 for both control and treatment groups at one and three weeks post-implantation, with a single exception of detectable IL-6 levels in the seven-day bioink with microsensors group. However, by three weeks post-implantation, these levels had decreased to undetectable levels (Figure 7B). Next, H&E histological analyses were performed to address the implanted grafts. In the two bioink groups, pink staining of the graft’s collagen was visible at both time points. Cell migration towards both the injected grafts and the alginate was seen after one week and further intensified after three weeks. Similar cellularization was seen in the alginate and psECM bioink groups, which was lower than the cellularization in grafts with microsensors (Figure 7C). To understand whether the high cellularization of the grafts was due to tissue integration or an immune response, immunostaining was performed for the F4/80 macrophage marker. Figure 7D revealed the presence of macrophages in the injection area as part of the cell migration process. While more macrophages were seen in the alginate group compared to the bioink ones in the first week, higher levels of macrophages were also seen in the group of bioink with microsensors after three weeks, consistent with the observed cellularization (Figure 7C).

## 4. Discussion

Skin grafts for the treatment of diabetic ulcers ideally aim to initiate tissue regeneration through structural support, relevant biochemical cues, and recruitment of relevant cells. By choosing psECM as the graft biomaterial, we create a rich microenvironment that mimics the complex biochemical profile of native skin tissue, thereby facilitating the initiation and promotion of regenerative processes and cell recruitment. To develop a psECM-based graft, we primarily established a decellularization protocol that effectively removed cellular components while preserving the essential ECM fibrous network structure and bioactivity. The minimal DNA content (<0.5 ng per mg dry tissue) achieved is consistent with established criteria for effective decellularization [48,49]. Effective removal of cellular material is critical for reducing immunogenicity and inflammation. Conversely, preservation of ECM structure and composition ensures retention of tissue-specific bioactivity and provides a scaffold conducive to tissue integration and remodeling [50,51,52].

To achieve a perfect structural fit between the graft and the wound, a 3D printing approach was employed. We hypothesized that by 3D scanning the wound, a corresponding graft could be printed to precisely match the wound shape. The resulting direct contact between the graft and the wound could thus facilitate the continuous transfer of nutrients, cells, oxygen, and signaling molecules. To enable graft fabrication via 3D printing, a psECM-based bioink was produced through enzymatic digestion. The resulting hydrogels, prepared at varying concentrations, exhibited weak gel behavior and underwent thermally induced gelation at physiological temperature—characteristics consistent with ECM-based bioinks reported in the literature and considered essential for 3D bioprinting applications [34,50,51]. Although no clear plateau in storage modulus (G′) was reached during the monitored gelation period, this behavior is in line with prior studies on collagen- and ECM-derived hydrogels, which often continue to undergo molecular rearrangement and network stabilization well beyond the initial sol–gel transition [53,54,55]. With the complementary use of a gellan gum support bath, these properties allowed precise deposition and maintenance of structural integrity postprinting, enabling 3D bioprinting of complex physiologically relevant shapes and personalized wound grafts.

A major obstacle in wound healing is providing regenerating tissue with sufficient oxygen to support biological processes. Oxygen enrichment therapies, such as hyperbaric therapy, are sometimes used; however, no practical clinical method currently exists to evaluate their success and monitor oxygen levels within the wound. In the current work, the approach of incorporating microsensors for ESR-based oxygen measurement within personalized graft was introduced to allow routine monitoring of oxygen levels. The ability to measure oxygen within psECM-printed graft was addressed using our recently developed handheld electron spin resonance scanner [34] and 5 mm × 5 mm × 5 mm printed models integrated with LiNC-BuO oxygen microsensors at depths of 0.5 mm and 1 mm from the surface under different oxygen levels. The clear dependence of ESR probe relaxation times on oxygen concentration confirmed the ability to measure oxygen levels in the skin graft through negative correlation, as previously demonstrated for ESR-based oximetry in different tissues [56,57]. Furthermore, better readings were obtained when microsensors were placed closer to the surface, due to the distance-dependent decline in ESR signal—also shown in previous publications [45,58,59]—with 0.5 mm distance being able to provide a reliable signal. It should be noted that while the signal at a larger depth of 1 mm was lower and thus less reliable, this can be compensated for by increasing the measurement time, which in typical hyperbaric chamber can reach up to 30 min (compared with the 2 min used in this work), which would translate to an increase in signal-to-noise-ratio by a factor of ~4. Further improvements in instrumentation and scanner design can result in acquiring signals at greater depths, up to 2 mm.

Altogether, this capability to integrate oxygen microsensors within the 3D-bioprinted graft addresses a critical gap in current wound care practices by allowing for real-time, non-invasive measurement of oxygen levels within the wound bed. The ability to monitor tissue oxygenation could provide valuable insights into the wound healing process and guide treatment decisions.

Upon incorporating LiNC-BuO oxygen microsensors within the psECM bioink, the combined graft was evaluated in terms of its cytocompatibility and biocompatibility to confirm that the graft’s capability to monitor oxygen levels does not compromise cell–graft interactions and, consequently, its pro-regenerative bioactivity. By enabling the adherence, survival, and proliferation of a keratinocyte–fibroblast co-culture, our combined graft demonstrated promising cytocompatibility when integrating up to 10 mM LiNC-BuO oxygen microsensors.

In terms of biocompatibility, although in vitro results revealed no immunogenic potential, our in vivo results showed a mild immune response following injection of psECM bioink integrated with oxygen microsensors, particularly macrophage infiltration and transient IL-6 elevation. In these studies, the oxygen microsensors were homogeneously dispersed in the psECM bioink, resulting in direct contact with the tissue, which likely contributed to the observed immune response. Hence, in future applications, we propose a refined strategy in which the graft is bioprinted with a defined spatial configuration. For example, oxygen microsensors can be embedded at a distance of 0.25 mm within the graft core, while the outer surface facing the wound bed consists of sensor-free psECM bioink. This design could minimize direct contact between the microsensors and host tissue, potentially further reducing the obtained moderate response while preserving oxygen-sensing capability. Furthermore, while mild immunogenic potential can possibly lead to graft failure in some cases, increased vascularization and cell recruitment can also be advantageous for tissue oxygenation and the initiation of regenerative processes, respectively [52]. Future studies should therefore evaluate the efficacy of the combined graft in promoting wound healing in diabetic animal models and assess whether integrated LiNC-BuO oxygen microsensors contribute to rapid healing or hinder its biocompatibility. If graft biocompatibility is compromised, different approaches can be taken, such as coating microsensors with oxygen-permeable and bioinert polymers [46]. Other possible approaches include PEGylation or hydrogel microencapsulation to further mitigate host immune activation [60,61].

While our findings demonstrate the potential of the combined oxygen-sensing 3D-printed graft, future studies should address several limitations. Primarily, long-term in vivo studies are needed to assess graft efficacy in promoting complete wound healing. Further, the stability and functionality of the oxygen microsensors over periods usually required for wound healing should be evaluated. Moreover, the optimal concentration and spatial distribution of microsensors, as well as their potential effects on wound healing, should be investigated.

## 5. Conclusions

The current study presents a technological proof of concept for a multifunctional approach to diabetic ulcer treatment that addresses several complex challenges in the field. It allows for personalized wound care through 3D bioprinting of grafts tailored to individual wound geometries, enhances tissue regeneration using ECM as a natural scaffold for cell growth and repair, and improves wound assessment through real-time oxygen monitoring, thereby enabling informed decisions about interventions such as hyperbaric oxygen therapy. The presented research establishes the feasibility of (i) producing psECM-based bioink, (ii) 3D printing it into personalized grafts which incorporate oxygen microsensors, and (iii) detecting oxygen levels within the printed graft. Thus, these findings constitute the basis for more advanced pre-clinical studies addressing long-term graft efficacy.

## Data Availability

The original contributions presented in this study are included in the article/Supplementary Material. Further inquiries can be directed to the corresponding author.

## Supplementary Materials

The following supporting information can be downloaded at: https://www.mdpi.com/article/10.3390/jfb17010028/s1, Figure S1: Time-sweep analysis of 2.75% psECM bioink along a 60 min test. Figure S2: Gellan gum particle size using different methods of processing with bead beater. Using two large beads with three medium beads yielded the smallest particle size, which allowed better resolution of printing within the support bed. Figure S3: Mice weight over 21 days following implantation of psECM bioink, psECM bioink integrated with oxygen microsensors (10 mM), or alginate hydrogel as a negative control.

## Abbreviations

The following abbreviations are used in this manuscript:

ECM	Extracellular matrix
psECM	Porcine skin extracellular matrix
ESR	Electron spin resonance
GG	Gellan gum
EGCG	Epigallocatechin gallate
