# Supplementary material for: 3D Printing of Oxygen-Sensing ECM-Based Skin Graft for Personalized Treatment of Chronic Wounds—A Technological Proof of Concept
Source: J Funct Biomater. 2026 Jan 1;17(1):28. doi: 10.3390/jfb17010028 (PMC12842197; doi:10.3390/jfb17010028)
Supplement: Supplementary file 1 [file jfb-17-00028-s001.zip › jfb-4032147-supplementary.pdf]

# 3D-Printing of oxygen-sensing ECM-based skin graft for personalized treatment of chronic wounds

Yehonatan Zur[1], Rotem Hayam[1], Nir Almog[2] Inna Kovrigina[1], Limor Baruch[1], Aharon Blank[2], and Marcelle Machluf[1]

## Supplementary Materials

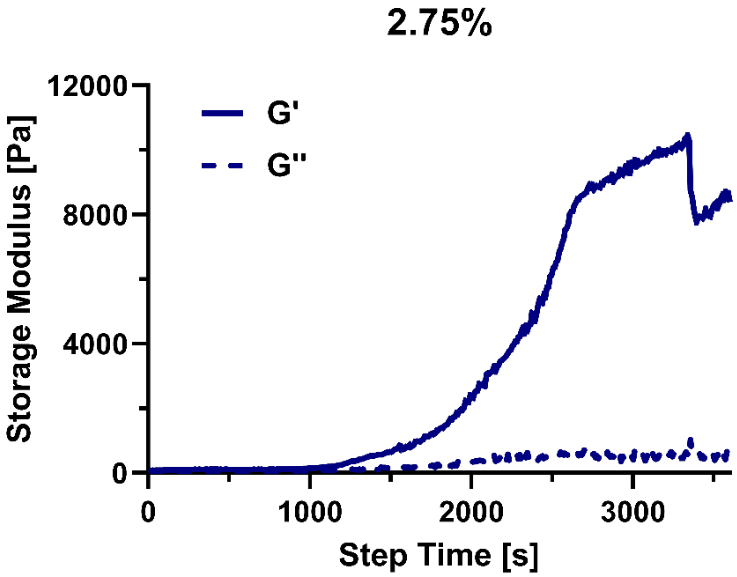


**Figure S1**: **Time Sweep analysis of 2.75% psECM bioink along a 60 minutes test.**

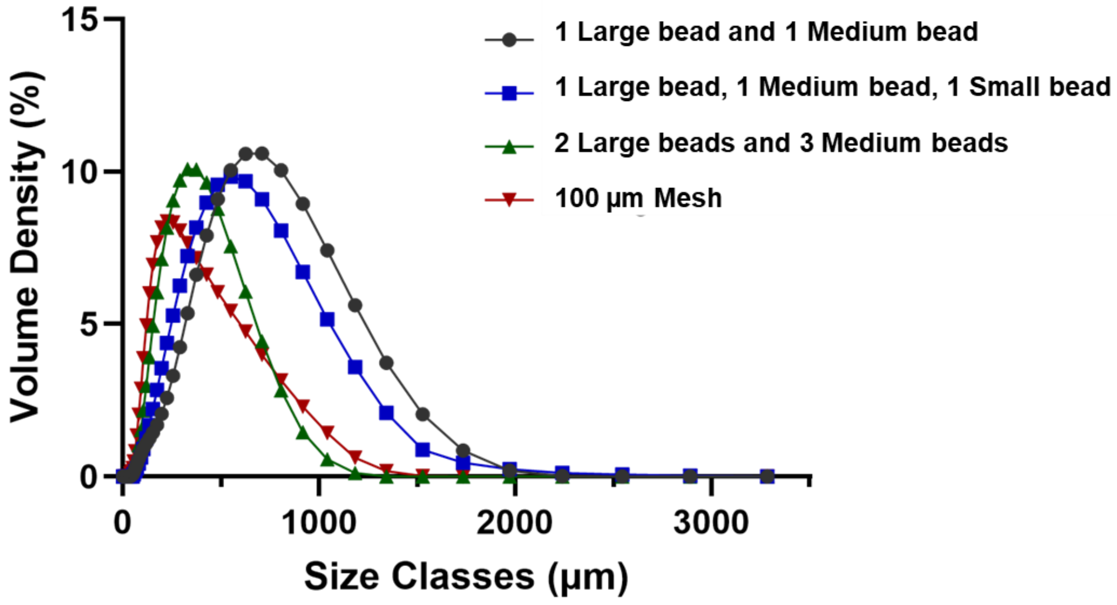


**Figure** S2: **Gellan gum particle size using different methods of processing with bead beater.** Using 2 large beads with 3 medium beads yielded the smallest particle size, which allowed better resolution of printing within the support bed.

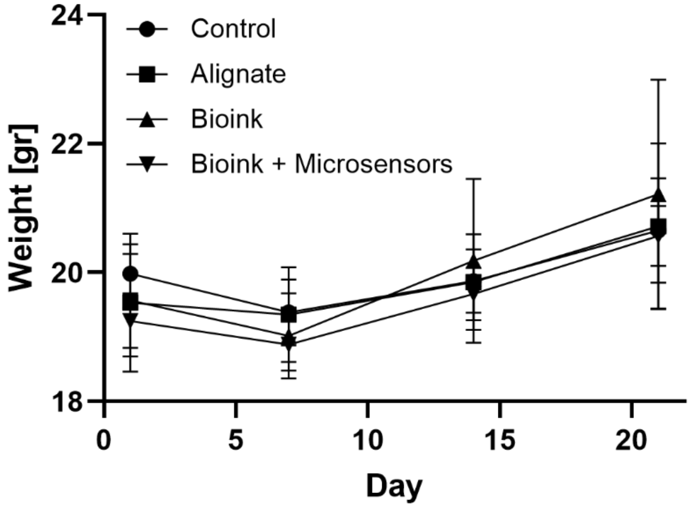


**Figure S3**: **Mice weight** along 21 days following implantation of psECM bioink, psECM bioink integrated with oxygen microsensors (10 mM) or alginate hydrogel as a negative control.
